# Cesarean rates according to the Robson classification: analysis in a municipal maternity in São Paulo

**DOI:** 10.31744/einstein_journal/2022AO0075

**Published:** 2022-07-08

**Authors:** Gabriela Guimarães Franco Ramos, Eduardo Zlotnik, Adolfo Wenjaw Liao

**Affiliations:** 1 Hospital Israelita Albert Einstein São Paulo SP Brazil Hospital Israelita Albert Einstein, São Paulo, SP, Brazil.; 2 Hospital Municipal da Vila Santa Catarina Dr. Gilson de Cássia Marques de Carvalho Hospital Israelita Albert Einstein São Paulo SP Brazil Hospital Municipal da Vila Santa Catarina Dr. Gilson de Cássia Marques de Carvalho; Hospital Israelita Albert Einstein, São Paulo, SP, Brazil.

**Keywords:** Cesarean section, Delivery, obstetric, Natural childbirth, Cesarean section, repeat, Delivery of health care

## Abstract

**Objective:**

To investigate the distribution of parturients at *Hospital Municipal da Vila Santa Catarina Dr. Gilson de Cássia Marques de Carvalho* according to the Robson classification, identify the cesarean rate in each Robson Group, and understand which group contributes more to the prevalence of Cesarean sections.

**Methods:**

This is a retrospective observational cross-sectional study conducted through the analysis of medical records of parturients admitted to *Hospital Municipal da Vila Santa Catarina Dr. Gilson de Cássia Marques de Carvalho* from October 2016 to August 2019.

**Results:**

A total of 9,794 births were recorded, and 31% were by Cesarean section. The most prevalent Robson Groups were Group 3 (25.7%-2,519), 1 (22.8%-2,234), and 5 (20.5%-2,006). The relative contribution of Cesarean sections was greatest in Groups 5 (39%), 2 (18%), and 1 (12.5%).

**Conclusion:**

This study demonstrated the Robson classification is useful to lead to a more critical view, identifying the groups that deserve more attention, since they are the major contributors to cesarean rates; hence, the management protocols could be modified aim to reduce cesarean rates.

## INTRODUCTION

Cesarean rates have been constantly increasing worldwide, and Cesarean section (C-section) is the most frequent surgery in developed countries.^(1)^ However, in 2015, data from each country were analyzed and the World Health Organization (WHO) concluded cesarean rates greater than 10% are not associated with a reduction in maternal or neonatal mortality. Thus, a concentrated effort must be made to ensure C-sections are performed only when really necessary.^(2)^

In 2018, Boerma et al. reported Brazil ranks second in C-section rates in the world, with 56%, after the Dominican Republic, with 58.1%.^(3)^ Such rates demonstrate the importance of assessing the delivery mode outcomes. According to the Ministry of Health in Brazil, the cesarean rate was 57.22%, in 2020, considering only births at public healthcare facilities.^(4)^ In the private sector, a much more critical scenario was observed, with 83.2% of deliveries performed by C-section, as reported by the National Regulatory Agency for Private Health Insurance and Plans (ANS - *Agência Nacional de Saúde Suplementar*).^(5)^

When performed based on appropriate obstetric indications, C-sections can reduce maternal and perinatal mortality. But there is no evidence it is beneficial to perform this surgery when there is no formal indication; on the contrary, like any other surgery, C-sections pose immediate and long-term risks.^(6)^ According to the American College of Obstetricians and Gynecologists (ACOG), the most common indications for the first cesarean are, in order of frequency, dystocia, abnormal fetal heart rate, anomalous fetal presentation, multiple pregnancy, and suspected fetal macrosomia.^(7)^ In Brazil, there are few studies on indications for C-sections, but an analysis of 2,441 deliveries performed at a maternity in the city or state of São Paulo showed a similar trend, and the main indications were fetal distress (23.2%), labor arrest (16.1%) and dystocia (13.9%).^(8)^

Since 2015, the WHO has proposed the Robson classification be used as a standard instrument worldwide, to assess, monitor and compare the rates and indications for C-sections over time at the same hospital, and among diverse hospitals,^(2)^ since this is an important strategy to categorize pregnant women and study the rates of vaginal and cesarean deliveries applicable to each group. For example, a study carried out in 13 maternity hospitals in France showed the most important contributors to the overall cesarean rate were Robson Groups 1, 2 and 5, accounting for 14.3%, 16.7% and 32.1% of C-sections performed, respectively.^(1)^ In Brazil, the *Universidade Federal do Ceará* reported a cesarean rate of 53.7% in its teaching maternity, and Robson Group 5 was primarily responsible for the high rate, at 25.2%, followed by Group 2, at 18.6%.^(9)^

Given the maternal and perinatal morbidity and mortality associated with this surgical procedure, there is a need to implement interventions aimed to refrain and reverse this current trend of constant growth in cesarean rates.^(1)^ Another important factor is costs generated, since C-sections incur significant additional expenses to healthcare systems, which are already overstretched and often present *deficits*, and play a relevant role in the financial sector worldwide.^(10,11)^

Data on the literature emphasize the importance of combating performance of the first cesarean in patients classified in Robson Groups 1 to 4. This would directly result in reducing the size of Group 5 and, consequently, in a relative contribution to the total cesarean rate.^(12)^ We must then apply the Robson classification to better understand our population and study the prevalence of C-sections, aiming to intervene in groups of pregnant women who have no real indications for this surgical procedure.

## OBJECTIVE

To investigate the distribution of parturients at *Hospital Municipal da Vila Santa Catarina Dr. Gilson de Cássia Marques de Carvalho* according to the Robson classification, identify the cesarean rate in each Robson Group, and understand which group contributes more to the prevalence of C-sections.

## METHODS

### Data sources and study population

This is a retrospective cross-sectional observational study, carried out through review and analysis of the medical records of pregnant women hospitalized for childbirth at the maternity of *Hospital Municipal da Vila Santa Catarina Dr. Gilson de Cássia Marques de Carvalho* (HMVSC), from October 2016 to August 2019.

This study was approved by the Research Ethics Committee of *Hospital Israelita Albert Einstein* (HIAE), (# 4.770.355, CAAE: 02091418.0.0000.0071).

**Figure 1:**
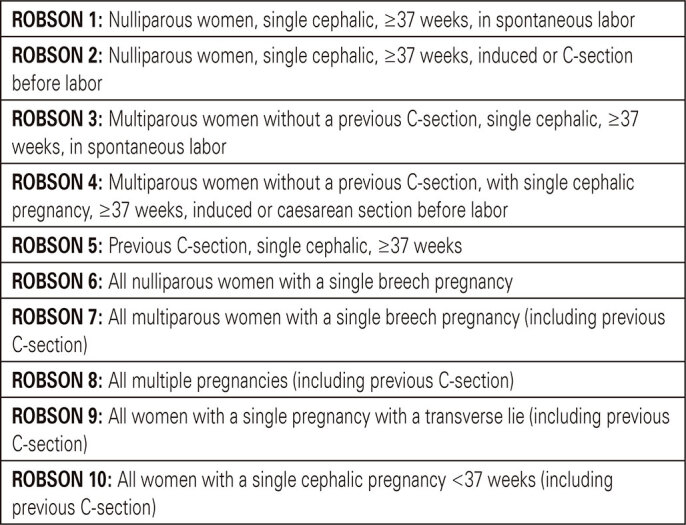
Robson classification

Source: adaptation from World Health Organization (WHO). Human Reproduction Programme (HRP). WHO statement on caesarean section rates. Geneva: WHO; 2015 [cited 2022 May 25]. Available from: https://apps.who.int/iris/bitstream/handle/10665/161442/WHO_RHR_15.02_eng.pdf^(2)^

Initially, the following items described in the medical records were identified: obstetric history (nulliparous or multiparous, previous C-section or not), number of fetuses (single or multiple pregnancy), fetal presentation (cephalic, breech or transverse), onset of labor (spontaneous or induced) and gestational age at the time of delivery. Next, the pregnant women were classified according to ten groups of Robson classification described in figure 1, and the prevalence of each group was evaluated by calculating its relative size (number of births in the group/total number of births), to obtain a profile of the patients seen at HMVSC.

Afterwards, the cesarean delivery rates were evaluated in each group, by calculating (number of C-sections/number of deliveries as per Robson Group). The relative contribution of cesarean delivery rates in each of these groups was calculated as number of C-sections in the group/total number of C-sections).

Cases of abortion (birth weight less than 500g and gestational age less than 20 weeks) were excluded from the study.

### Statistical analysis

The sample was characterized from the mean, standard deviation and median for quantitative variables, and relative and absolute frequencies for qualitative variables.

Analyses were performed using the SPSS, version 26.0 and a significance level of 5% was adopted.

## RESULTS

### Sample characteristics

In the period from October 2016 to August 2019, 9,794 births were recorded from 9,356 women, considering 16 of them had 3 deliveries during the period, 406 had 2, and 8,934 patients had only 1 delivery. Regarding the maternal characteristics, the mean age was 27 years old, ranging between 13 and 47 years; 1,037 (10.8%) were adolescents (aged less than or equal to 18 years old), and 373 (0.4%) were aged 40 years or more. Most mothers declared themselves black (52.0%-3,529) or white (32.2%-2,184) and had completed (45.6%-3,095) or not high school (22.5%-1,526).

There were 9,928 newborns, but not all had complete information in their medical record. As for their condition at birth, 98.9% (9,800) were liveborn and 1.1% (110) stillborn. The mean weight was 3,140g, standard deviation of 619g.

Valid information regarding demographics is presented in table 1.

**Table 1:** Demographic data

Demographic data	
Maternal age (n=9,630), n (%)	
Adolescent (≤18 years old)	1,037 (10.8)
≥40 years old	373 (0.4)
Skin color (n=6,790)	
White	2,184 (32.2)
Black	3,529 (52.0)
Pardo	1,031 (15.1)
Other	46 (0.7)
Education (n=6,786), n (%)	
Illiterate	8 (0.1)
Incomplete Elementary	776 (11.4)
Complete Elementary	621 (9.2)
Incomplete High School	1,526 (22.5)
Full Medium	3,095 (45.6)
Incomplete Higher	359 (5.3)
University Degree (graduate)	401 (5.9)
Condition at birth (n=9,910), n (%)	
Liveborn	9,800 (98.9)
Stillborn	110 (1.1)
Birthweight (n=9,858)	
Mean and standard deviation	3,140g±619g

The characteristics of pregnancies and births are shown in table 2.

**Table 2:** Characteristics of pregnancies and births

	n (%)
Type of pregnancy	
Single	9,660 (98.6)
Multiple	134 (1.4)
Type of fetal presentation (n=9,755)	
Cephalic	9,374 (96.1)
Transverse	35 (0.4)
Breech	346 (3.5)
Gestational age	
Preterm (<37 weeks)	917 (9.4)
Term (≥37 weeks)	8,877 (90.6)
Delivery	
Cesarean	3,036 (31.0)
Vaginal	6,674 (68.1)
Forceps	84 (0.9)

### Robson classification and mode of delivery

As shown in table 3, the most prevalent Robson classifications in the service were Group 3 (25.7%-2,519) including multiparous women without a full-term pregnancy and no previous C-section, single fetus with a cephalic presentation, and spontaneous labor; Group 1 (22.8%-2,234), which refers to nulliparous women with a full-term pregnancy, single fetus with a cephalic presentation, and spontaneous labor; and Group 5 (20.5%-2,006), comprising multiparous women who came to term and had a single fetus with a cephalic presentation, but underwent at least one C-section. Analyzing the birth rates within each group, the groups with the highest prevalence rates of C-sections were Group 9 (100%-32), with fetuses in transverse lie; Group 8 (89.47%-119) comprising women with multiple pregnancies; Group 7 (87.17%-119), pregnant women without a previous cesarean who had a breech presentation; and Group 6 (86.13%-163), which also includes fetus in breech presentation, but no previous cesarean.

**Table 3:** Robson classification and mode of delivery

Robson classification	Number of deliveries	Relative size of group^†^ (%)	Number of cesarean deliveries in each group	C-section rate in each group^‡^ (%)	Relative contribution to overall C-section rate^#^ * (%)
1. Nulliparous women, single cephalic, ≥37 weeks, in spontaneous labor	2,234	22.80	381	17.05	12.55
2. Nulliparous women, single cephalic, ≥37 weeks, induced or C-section before labor	1,182	12.10	547	46.28	18.02
3. Multiparous women without a previous C-section, single cephalic, ≥37 weeks, in spontaneous labor	2,519	25.70	106	4.21	3.49
4. Multiparous women without a previous C-section, with single cephalic pregnancy, ≥37 weeks, induced or C-section before labor	653	6.70	148	22.66	4.87
5. Previous C-section, single cephalic, ≥37 weeks	2,006	20.50	1,184	59.02	39.00
6. All nulliparous women with a single breech pregnancy	137	1.40	118	86.13	3.89
7. All multiparous women with a single breech pregnancy (including previous C-section)	187	1.90	163	87.17	5.37
8. All multiple pregnancies (including previous C-section)	133	1.40	119	89.47	3.92
9. All women with a single pregnancy with a transverse lie (including previous C-section)	32	0.30	32	100.00	1.05
10. All women with a single cephalic pregnancy <37 weeks (including previous C-section)	711	7.30	238	33.47	7.84
Total births	9,794	100	3,036	-	100

^†^ Relative size of group (number of deliveries in the group)/(total number of deliveries); ^‡^ Cesarean section rate (%) in each group (number of C-sections/number of deliveries in each group); ^#^ Relative (%) contribution on the overall C-section rate (number of cesarean deliveries in the group)/(total number of cesarean deliveries); * χ^2^; p<0.001 refers to the relative contribution of cesarean rates.

The relative contribution of C-section deliveries was greater in Group 5, with 1184 deliveries accounting for 39% of procedures; Group 2, with 547 deliveries (18%), comprising nulliparous women with a cephalic fetus, no spontaneous labor; and Group 1 with 381 (12.5%) deliveries. Pregnant women classified in Robson Groups 1 to 4 accounted for 67.3% (6,588) of our sample, and for 38.9% (1,182) of C-sections.

## DISCUSSION

In the present study, the most prevalent Robson classifications observed at HMVSC were Group 3 (25.7%), Group 1 (22.8%), and Group 5 (20.5%). These data were slightly different from those published by the Live Birth Information System (SINASC - *Sistema de Informações sobre Nascidos Vivos*),^(13)^which indicated, in 2019, out of 2,848,721 live births in Brazil, most were in the Robson 5 (23.42%), 3 (19.59%) and 1 (17.63%) Groups. In the study population, Groups 1 and 3 were dominant, comprising women who had spontaneous labor, as opposed to the Brazilian average, in Robson Group 5, consisting of patients with a previous C-section, which stands out. This is probably because we have a protocol that allows entering data in a later period, thus increasing the likelihood of patients in spontaneous labor.

In our sample of 9,794 deliveries, the C-section rate was 31%, which is below the Brazilian rate of 41.9% for public healthcare facilities in 2017, according to the Ministry of Health.^(4)^ However, both rates are still above those recommended by the WHO; the organization stated C-sections rates greater than 10% are not associated with reduction in maternal and neonatal mortality.^(2)^

Of 1,604,515 C-sections (55.82% of births) performed in Brazil, in 2019, the patients were primarily in Robson Group 5 (35.4% of live births), Group 2 (17%) and Group 1 (13.79%),^(13)^ which were the most important contributors.^(1,9,13)^In our study, the same trend was observed - Robson Group 5 with 39% (1,184) of C-sections, Group 2 with 18% (547), and Group 1 with 12.5% (381).

A slightly different result was demonstrated in a study analyzing deliveries performed in Brazil, between 2011 and 2012.^(14)^Although the distribution of pregnant women in the Robson Groups was similar to ours, the groups with the greatest impact on the C-section rate, both at public and private facilities, were 2, 5 and 10. Group 10 comprises premature fetuses and accounted for approximately 10% of C-sections performed in the country during this period, ranking third. In the present study, this group accounted for 7.8% of C-sections, after Group 1, with 12.5%. In other words, more C-sections were observed in nulliparous patients with a full-term fetus in cephalic presentation, than in premature ones.

The main arguments for performing a C-section in pre-term fetuses would be decreased fetal metabolic reserves during labor or fear of birth trauma; however, no study has suggested that C-sections improve neonatal outcome in cases of spontaneous pre-term labor.^(15)^

One possible explanation for the difference observed between the studies is that in our service we reject the idea that prematurity *per se* is an indication for C-section. The protocols used in other services evaluated by this Brazilian study may be more permissive regarding the indications for delivery in the setting of prematurity.^(14)^

In the same study,^(14)^ pregnant women classified in Robson Groups 1 to 4, who are parturients without a previous cesarean, full-term and cephalic presentation, that is, patients in a favorable condition for vaginal delivery, accounted for 68.1% of pregnant women and for 49.4% of C-sections performed. In the present study sample, Groups 1 to 4 accounted for 67.3% (6,588) of pregnant women and for 38.9% (1,182) of C-sections carried out in the service; this demonstrated that, despite representing a significant percentage of the cesarean deliveries in these groups, more vaginal deliveries were reported, as compared to other public services in the country.

Robson Group 5 accounted for 39% (1,184) of C-sections, a significant percentage of patients who had a previous C-section and underwent the same procedure again. This is like a “domino effect”, considering the rise in cesarean rates in nulliparous women subsequently increases the number of patients with a previous C-section and the probability of undergoing the same surgery. In fact, a study carried out by WHO compared cesarean rates according to Robson classification in 21 countries, between the periods 2004 to 2008 and 2010 to 2011, and showed cesarean rates increased over time, rising the absolute contribution to cesarean rates in Group 5.^(16)^

Here, we have an important factor that may change in the long run: as the literature on Robson Groups indicates, we must intervene in to reduce the cesarean rates,^(1,9,12,14,16)^ by combatting the first cesarean in pregnant women classified as Robson 1 to 4 (pregnant women who have never had a cesarean, have a full-term fetus, and a cephalic presentation). In other words, by establishing better defined protocols on the indication of “acute fetal distress”, we would observe a direct decrease in the relative size of Group 5, leading to lower prevalence of patients with previous C-sections; therefore, we would achieve a relative decrease in total cesarean rate. In addition, the induction of labor and its proper management would be an important measure to reduce cesarean rates globally, especially in Group 5, as encouraging vaginal delivery after a cesarean could have a strong impact, decreasing the number of iterative patients in the long run.^(17)^In another strategy,^(17)^ induction failure could be diagnosed only if the patient did not enter the active phase of labor after 12 hours of oxytocin use, with premature rupture of ovular membrane. Therefore, this corroborates the WHO statement, *i.e.*, the interpretation of Robson classification in each service can optimize the use of C-sections by identifying, analyzing and focusing resources on specific groups that are relevant.^(2)^

According to ACOG recommendations for safe prevention of primary cesarean delivery,^(7)^ operative vaginal delivery in the second stage of labor by experienced and well trained physicians should be considered a safe, acceptable alternative to cesarean delivery. In our hospital, only 0.9% were operative vaginal delivery - only forceps because we have no vacuum extractor available in our service. Hence, training obstetricians and residents to perform operative vaginal deliveries would also contribute to a safe drop in cesarean rates.

## CONCLUSION

This study demonstrated the Robson classification is really useful to map the local profile of each maternity hospital and adopt a more critical view of our obstetric practices, identifying the groups that deserve more attention, since they are the largest contributors to C-section rates.
